# Carvedilol, an Adrenergic Blocker, Suppresses Melanin Synthesis by Inhibiting the cAMP/CREB Signaling Pathway in Human Melanocytes and Ex Vivo Human Skin Culture

**DOI:** 10.3390/ijms21228796

**Published:** 2020-11-20

**Authors:** Myoung Eun Choi, Hanju Yoo, Ha-Ri Lee, Ik Joon Moon, Woo Jin Lee, Youngsup Song, Sung Eun Chang

**Affiliations:** 1 Department of Dermatology, Asan Medical Center, University of Ulsan College of Medicine, 88 Olympic-ro 43-gil, Songpa-gu, Seoul 05505, Korea; mechoi316@naver.com (M.E.C.); julia_yoo@hanmail.net (H.Y.); ikjun.moon@gmail.com (I.J.M.); uucm79@hanmail.net (W.J.L.); 2 Bio-Medical Institute of Technology (BMIT), University of Ulsan College of Medicine, Ulsan 05505, Korea; mku0_0@naver.com; 3 Department of Biomedical Sciences, Asan Medical Center, University of Ulsan College of Medicine, 88 Olympic-ro 43-gil, Songpa-gu, Seoul 05505, Korea

**Keywords:** carvedilol, adrenergic blocker, melanin synthesis, cAMP/CREB signaling

## Abstract

Catecholamines function via G protein-coupled receptors, triggering an increase in intracellular levels of 3′,5′-cyclic adenosine monophosphate (cAMP) in various cells. Catecholamine biosynthesis and the β-adrenergic receptor exist in melanocytes; thus, catecholamines may play critical roles in skin pigmentation. However, their action and mechanisms mediating melanogenesis in human skin have not yet been investigated. Therefore, we examined the potential anti-melanogenetic effect of carvedilol, a nonselective β-blocker with weak α1-blocking activities. Carvedilol reduced melanin content and cellular tyrosinase activity without compromising cellular viability in normal human melanocytes as well as in mel-Ab immortalized mouse melanocytes. Carvedilol downregulated microphthalmia-associated transcription factor (MITF), tyrosinase, tyrosinase-related protein (TRP)-1, and TRP-2. Carvedilol treatment led to the downregulation of phosphor-cAMP response element-binding protein (CREB). Moreover, the increase in cAMP levels upon treatment with forskolin reversed the anti-melanogenic action of carvedilol. In addition, carvedilol remarkably reduced the melanin index in ultraviolet-irradiated human skin cultures. Taken together, our results indicate that carvedilol effectively suppresses melanogenesis in human melanocytes and ex vivo human skin by inhibiting cAMP/protein kinase A/CREB signaling. The anti-melanogenic effects of carvedilol have potential significance for skin whitening agents.

## 1. Introduction

A wide range of pigmentary skin disorders have a significant psychological and social impact on patients. Various treatment modalities, including systemic and topical agents as well as laser therapy, have been developed [1,2,3]. However, the results are often unsatisfactory and adverse reactions, such as post-inflammatory hyperpigmentation (PIH) and hypopigmentation, from treatment are common. Additionally, treatment is expensive and time-consuming [4,5,6].

Catecholamines, which include dopamine, epinephrine, and norepinephrine, are signaling molecules that act as neurotransmitters and endocrine hormones. In the skin, the biosynthesis and degradation of catecholamines occur in human keratinocytes, but catecholamine synthesis in melanocytes is somewhat different [7,8,9]. Catecholamines function via G protein-coupled receptors (GPCRs). The binding of catecholamines to the GPCRs triggers the activation of intracellular adenylate cyclase, which synthesizes 3′,5′-cyclic adenosine monophosphate (cAMP) from ATP [10]. The second messenger cAMP exerts its activity by binding the R-subunit of protein kinase A (PKA), resulting in the phosphorylation of cAMP response element-binding protein (CREB). GPCRs are activated by amines and peptides, including glucagon, parathyroid hormone, secretin, and calcitonin [10].

Adrenergic receptor antagonists include α-receptor and β-receptor antagonists. α-Receptor antagonists are subcategorized into non-selective, α_1_-selective, and α_2_-selective agents, whereas β-receptor antagonists are subclassified as non-selective, β_1_-selective, and β_2_-selective agents based on their selective blocking activities. Unlike first-generation non-selective β-receptor antagonists, such as propranolol, timolol, and nadolol, carvedilol is a third-generation non-selective β-blocker that displays vasodilator actions by blocking α_1_-adrenoreceptors (α_1_-ARs) [11]. Therefore, carvedilol is a nonselective β-blocker with weak α1-blocking activities [12]. It is mainly used as an oral medication to control high blood pressure and congestive heart disease, similar to other β-blockers [12]. However, third-generation β-blockers exhibit angiogenic, antioxidant, anti-proliferative, anti-hypertrophic, and anti-apoptotic activities that require further elucidation [11]. In the dermatologic field, due to its antioxidant and anti-inflammatory actions, carvedilol is often used in oral formulations to treat erythematotelangiectatic rosacea [13,14]. Moreover, the antioxidant activity of carvedilol results in the prevention of ultraviolet (UV)-induced skin carcinogenesis, making it an attractive agent for managing UV-associated skin diseases [15,16,17,18]. However, the chemopreventive effects of carvedilol are not mediated directly through ARs. [19] Although the exact mechanism is relatively unknown, the cAMP/PKA and PKC-δ signaling pathway could be related to the properties of carvedilol against skin metastasis [20].

In the early stages of investigating pigmentation, human melanocytes were found to express α-1-AR signaling after extracellular induction with norepinephrine. However, β-ARs were not found after stimulation with adrenergic signaling in melanocytes [8]. Conversely, Cillbro et al. later demonstrated that a specific functional β_2_-AR signal exists in human melanocytes and that β_2_-AR stimulation leads to pigmentation through the β_2_-AR/cAMP pathway [7]. Therefore, the role of catecholamines in the control of pigmentation was suggested, and cAMP is considered the main axis for the catecholamine control of melanogenesis.

Melanogenesis is a complex process that involves numerous pathways. Tyrosinase, tyrosinase-related protein 1 (TRP-1), and TRP-2, also called dopachrome tautomerase (DCT), are the three main melanocyte-specific enzymes involved in melanin synthesis [21]. Melanogenesis is induced or inhibited by numerous factors, including hormones, cytokines, neurotransmitters, growth factors, and micromolecules [21,22,23].

The most important positive regulator is the melanocortin-1 receptor and its ligands, melanocortins and adrenocorticotropic hormone [23]. However, miscellaneous factors involved in melanogenesis are β-endorphin, estrogens, androgens, vitamin D_3_, and catecholamines [23]. Cumulative evidence has suggested that L-tyrosine and L-DOPA, which are substrates and intermediates of melanogenesis, act as inducers and positive regulators of the melanogenic pathway, in addition to regulators of other cellular functions [24]. Moreover, Jeff Howe et al. suggested that the induction and regulation of melanogenesis by L-tyrosine is mediated by a direct activation of adrenergic receptors by L-tyrosine, rather than caused by its metabolic products such as catecholamines [25]. In their studies, norepinephrine and epinephrine stimulated tyrosinase activity, but their inductive effect on melanin synthesis was comparatively lower than L-tyrosine [25].

Catecholamines may play important roles in the skin pigmentation system; however, their effects on melanogenesis in regard to the action of third-generation non-selective β-blocker have not yet been explored. Therefore, in the present study, we aimed to investigate whether carvedilol affects melanogenesis and explored its mechanisms of action in human melanocytes and ex vivo human skin and its potential use as a whitening product.

## 2. Results

### 2.1. Carvedilol Suppresses Melanogenesis

**Figure 1:**
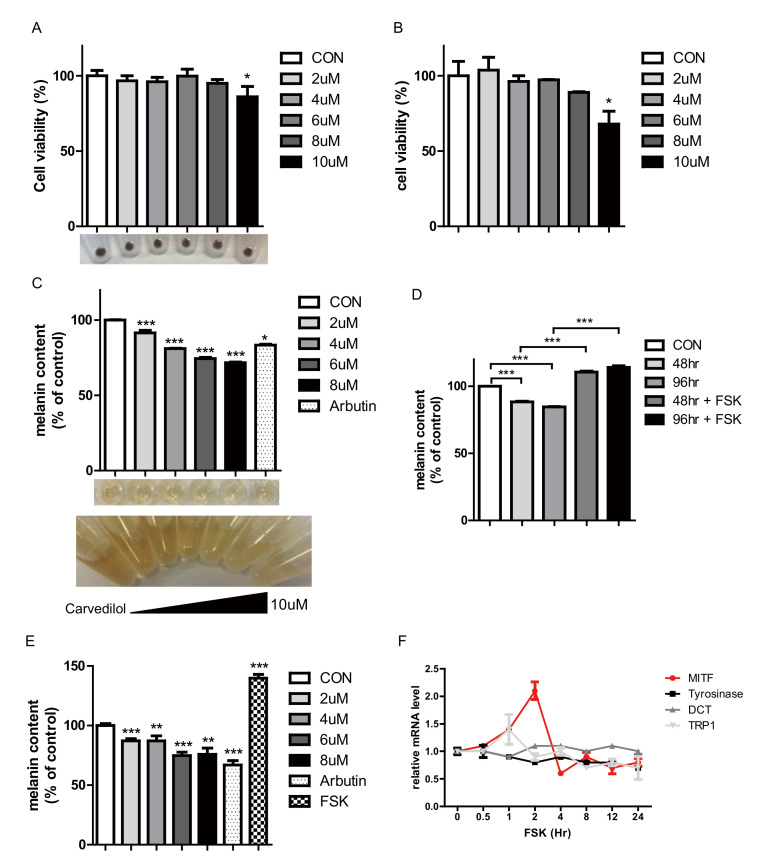
Effect of carvedilol on melanin production in normal human melanocytes (NHMs) and Mel-Ab cells without affecting cell viability. (**A**) Cell viability was measured using the WST assay in NHMs. NHMs were treated with 2–10 μM carvedilol for 5 days. (**B**) Cell viability was measured using the WST assay in Mel-ab cells. Mel-ab cells were treated with 2–10 μM carvedilol for 3 days. (**C**) Effect of carvedilol on the cellular melanin content when NHMs were treated with the indicated concentrations of carvedilol for 5 days. (**D**) Melanin content of NHMs at 48 and 96 h after treatment with vehicle control, 8 μM carvedilol, and carvedilol with 10 μM forskolin (FSK). (**E**) Melanin content of Mel-Ab cells treated with indicated concentrations of carvedilol for 72 h. (**F**) Time-dependent curve of mRNA levels of microphthalmia-associated transcription factor (*MITF*) in NHMs treated with 10 μM FSK for up to 24 h. FSK increased the transcription of *MITF* to the greatest degree at 2 h in NHMs, possibly via the cAMP/PKA/CREB pathway. Time-dependent curve of the mRNA levels of tyrosinase, *DCT*, and *TRP1* in NHMs treated with 10 μM FSK for up to 24 h. Data are expressed as means ± SD of three independent experiments. * *p* < 0.05, ** *p* < 0.01, *** *p* < 0.001 vs. controls.

The cytotoxicity of carvedilol against normal human melanocytes (NHMs) and Mel-ab cells was assessed by a WST cell proliferation assay. A carvedilol concentration of 10 μM began to show cytotoxicity against both NHMs and Mel-ab cells (Figure 1A,B). Therefore, in further assessments, we used 8 μM of carvedilol, which is not cytotoxic to NHMs.

Treatment with carvedilol decreased the melanin content in a dose-dependent manner without affecting the viability of NHMs (Figure 1C). The melanin content was decreased by 28.36% after 96 h of 8 μM carvedilol treatment (Figure 1C). The addition of 100 mg/mL arbutin reduced the melanin content to a lesser degree than carvedilol (Figure 1C). After 4 days of carvedilol treatment, the melanin content decreased in a time-dependent manner (Figure 1D). However, treatment with forskolin (FSK) for 4 days after pre-treatment with carvedilol induced an increase in melanin content (Figure 1E). FSK increases the transcription of *MITF* to the greatest degree at 2 h in NHMs, and is believed to function via the cAMP/PKA/CREB pathway (Figure 1F).

### 2.2. Carvedilol Inhibits the Expression of MITF and Its Target Genes and Decreases Phospho-CREB Levels in NHMs

**Figure 2:**
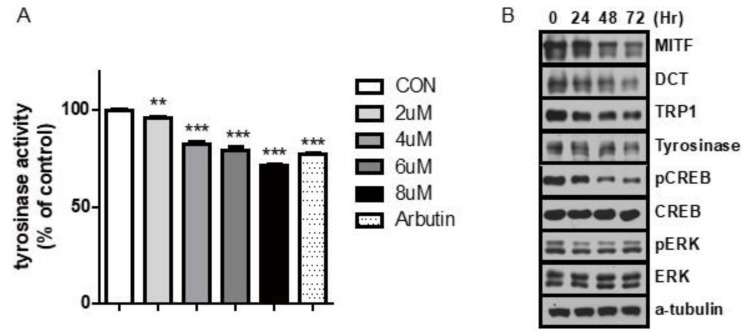
Effect of carvedilol on tyrosinase and melanogenesis-related protein expression. (**A**) Effect of carvedilol on tyrosinase activity when NHMs were treated with the indicated concentrations of carvedilol for 5 days. (**B**) Protein expression levels of genes associated with melanogenesis processes (MITF, DCT, TRP1, and tyrosinase) were examined using Western blotting. The assay for intracellular signaling of p-CREB and ERK revealed a decrease in p-CREB levels upon carvedilol treatment. The p-ERK level was not changed. Data are expressed as the means ± SD of three independent experiments. ** *p* < 0.01, *** *p* < 0.001 vs. controls.

Because carvedilol decreased melanin accumulation, we investigated cellular tyrosinase activity. Treatment with carvedilol decreased cellular tyrosinase activity in a dose-dependent manner in NHMs (Figure 2A). Tyrosinase activity decreased by 28.48% after 96 h of 8 μM carvedilol treatment (Figure 2A). We next determined whether carvedilol affects the expression of MITF, which plays a crucial role in the regulation of tyrosinase and downstream melanogenic genes. FSK treatment increased intracellular cAMP levels and reversed the anti-melanogenic actions of carvedilol. Carvedilol significantly reduced the protein levels of MITF, a central transcription factor of melanogenesis, at 72 h (Figure 2B). Furthermore, the expression of its target genes, such as tyrosinase and *TRP-1*, was decreased after carvedilol treatment (Figure 2B). These results indicate that carvedilol inhibits melanogenesis by downregulating MITF signaling.

Next, we investigated the intracellular signaling pathways of melanogenesis, which regulate *MITF* transcription, by measuring the expression levels of phospho-CREB and phospho-ERK. Phospho-ERK levels did not change over time following carvedilol treatment; however, phospho-CREB levels were decreased (Figure 2B). Consistent with previous observations, our results revealed that carvedilol inhibits melanogenesis by inhibiting the cAMP/PKA/CREB signaling pathway. Moreover, FSK treatment reversed the anti-melanogenic action of carvedilol by increasing cAMP levels.

### 2.3. Melanin Index and Immunohistochemical Staining in Ex Vivo Human Skin Culture

**Figure 3:**
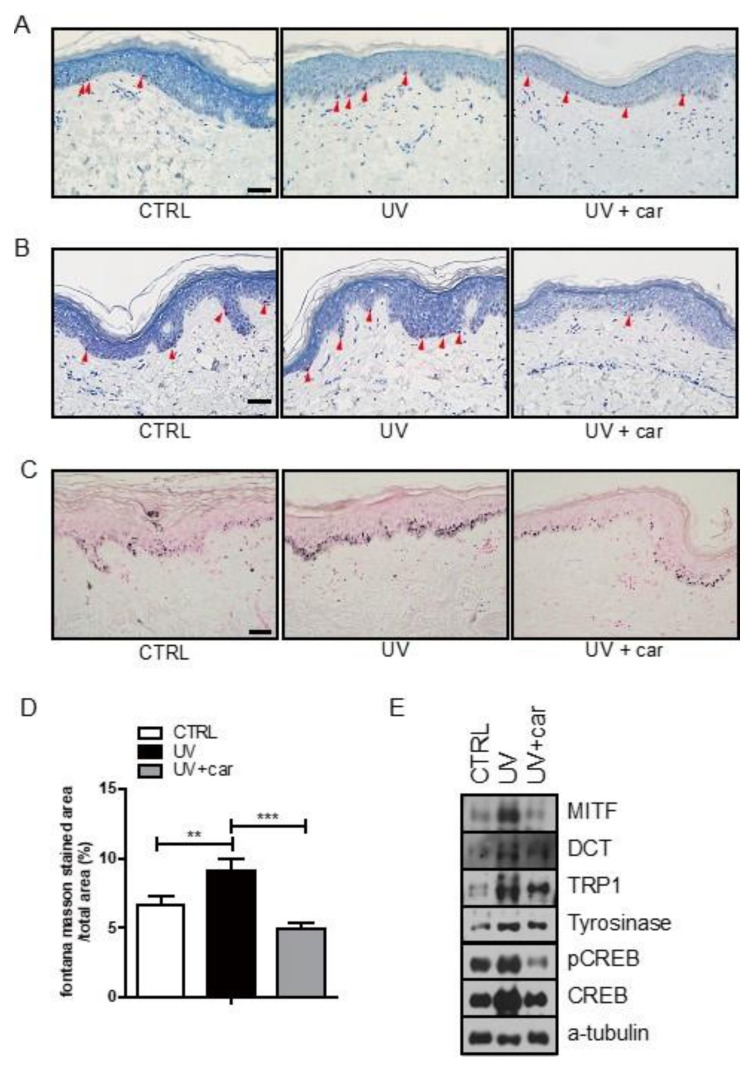
Carvedilol suppresses UVR-induced melanin accumulation in ex vivo human skin. (**A**) Microscopic images of Melan-A immunohistochemistry for melanocyte staining. Arrowheads indicate positive Melan-A staining (red color). Bar = 50 μm. (**B**) Microscopic images of HMB45 immunohistochemical staining for melanocytes. Arrowheads indicate positive HMB45 staining (red color) Bar = 50 μm. (**C**) Representative images of Fontana–Masson-stained paraffin-embedded sections treated with vehicle (control), UVB + vehicle, or UVB + carvedilol. Bars = 50 μm. (**D**) The ratio of the Fontana–Masson-stained area per total area for control, UVB, and UVB + carvedilol. (**E**) Cell lysates of control, UVB, and UVB + carvedilol were analyzed by Western blot using antibodies, MITF, DCT, TRP1, tyrosinase, and p-CREB, and total CREB and equal protein loading were confirmed by α-tubulin. Data are expressed as the means ± SD of three independent experiments. ** *p* < 0.01, *** *p* < 0.001 vs. controls.

The epidermal melanocyte density and melanin index in ex vivo human skin culture tissue sections were detected by Melan-A and Fontana–Masson’s staining, respectively. Carvedilol did not affect the number of Melan-A (+) melanocytes in the specimen treated with carvedilol + UV radiation (UVR) compared with that in the specimen treated with UVR alone (Figure 3A). HMB45(+) melanocytes may indicate that melanocytic activity increased upon UVR treatment and was reversely downregulated following carvedilol treatment (Figure 3B). However, melanin content was significantly reduced in specimens treated with carvedilol + UVR compared with that in UVR-alone treated specimens (Figure 3C). For calculating the melanin index, the fraction of Fontana–Masson’s stained area over the total area between a specimen exposed to UVR and that to carvedilol + UVR was calculated and compared (Figure 3D). Cell lysates of each specimen were analyzed by Western blot assay, which revealed that tyrosinase, TRP1, and DCT were increased by UVR and downregulated by carvedilol treatment (Figure 3E). As a result, carvedilol remarkably reduced the melanin index and melanogenesis-related proteins, showing its anti-melanogenic effect on UVR-treated human skin.

## 3. Discussion

Melanin is the pigment responsible for skin and hair color and is synthesized in melanosomes by melanocytes. Although epidermal melanin plays an important protective role against UVR, melanin overproduction and accumulation in the skin causes troublesome skin hyperpigmentary disorders, such as PIH, photoaging-associated dyspigmentation, melasma, and solar lentigines [18,26]. Therefore, the inhibition of melanogenesis has been the focus of medicinal and cosmetic treatments for skin beauty and health. Considerable efforts have been made to identify new and effective anti-pigmentation agents. However, the anti-melanogenesis mechanisms of the specific agents are currently uncertain and have generally been evaluated in mouse cells, which yield results that are not always consistent with those of human skin trials [27,28]. Moreover, as the melanogenesis of melanocytes is tightly regulated by keratinocytes and other neighboring cells, cocultured human cells or ex vivo human skin are more reliable experimental settings for the exploration of effective whitening agents [29]. Most skin whitening agents, whether naturally or chemically derived, may cause skin toxicity or irritation, which can be predicted to a certain extent using in vitro cellular viability assays with melanocytes. In our study, carvedilol did not show cytotoxicity when used in moderate doses.

Hydroquinone topical creams can result in undesired hypopigmentation disorders and skin toxicity [30,31,32]. Furthermore, some whitening cosmetics have disastrous consequences by inducing hypopigmentation through the degradation of tyrosinase proteins [33,34,35]. Whitening agents can be used at higher doses depending on the user to maximize the whitening of hyperpigmentary lesions. Therefore, efforts to discover safe and healthy skin whitening agents are continuously under exploration.

Therefore, the present study was designed to be conducted in normal human cells and ex vivo human skin. Furthermore, based on catecholamines’ established action mechanism of G signaling, which increases the cellular cAMP level, we believed that an adrenergic blocker could decrease cAMP levels and inhibit the UVR/cAMP/CREB signaling pathway, which is the main mechanism for UV-induced skin hyperpigmentation [36,37]. Therefore, we hypothesized that adrenergic blockers that decrease cAMP levels reduce melanin synthesis.

**Figure 4:**
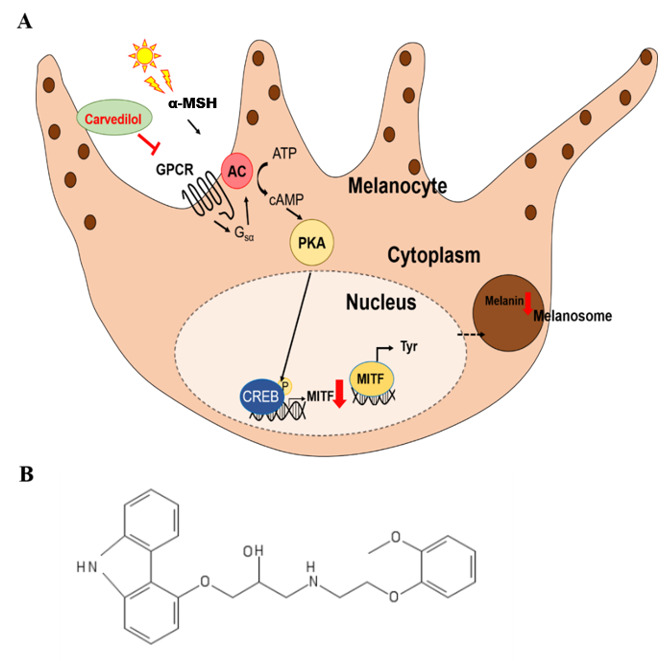
Summary of the mechanistic pathway by which carvedilol affects melanogenesis. (**A**) Summary of the mechanistic pathway underlying the effect of carvedilol on melanogenesis. (**B**) Chemical structure of carvedilol (1-(9*H*-carbazol-4-yloxy)-3-[2-(2-methoxyphenoxy) ethylamino] propan-2-ol). (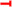: inhibition, 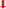: decrease).

In addition, to develop safe whitening agents, reliable and reproducible mechanisms of anti-melanogenesis should be pursued in parallel. The most physiologically significant stimulus is UV, and among UVR signaling to epidermal melanocytes, the CREB axis is the most established pathway for the regulation of melanogenesis in the human epidermis [29]. Exposure to UV successively activates cAMP production, PKA, and the transcription factor CREB, which, in turn, induces the expression of MITF and downstream target melanogenic genes [38,39]. In addition to CREB phosphorylation by PKA, recent studies have demonstrated that the recruitment of CREB-regulated transcription coactivator (CRTC) 3 to the CREB transcription complex is also required for cAMP-stimulated MITF. MITF performs the most essential role in the regulation of melanin synthesis and the resultant transcription of melanogenic enzymes [26,40,41]. During this intracellular signaling process, melanogenesis is regulated by a key enzyme, tyrosinase, and additional enzymatic proteins, such as TRP-1 and DCT [1,2,3,4]. In the present study, carvedilol effectively reduced the phosphorylation of CREB, which indicates that it reduced MITF and tyrosinase proteins by inhibiting *MITF* transcription (Figure 4). Considering that *MITF* mRNA gene regulation is intricately controlled and rescued by other intracellular signaling molecules and coactivators, the transcriptional level regulation of *MITF* is a promising strategy for exploring healthy skin whitening ingredients because the survival function of MITF is preserved and rescued [1,38,40]. Indeed, when we investigated FSK-induced *MITF* transcription, *MITF* mRNA was found to have its own peak response curve for melanogenesis and cellular survival for cellular homeostasis. The biological functions of melanocytes seemed to be intrinsically regulated by other feedback signals in human melanocytes. Moreover, carvedilol has a lower risk of adverse events of hypopigmentation as it attenuates cellular tyrosinase activity over time, rather than abruptly.

Catecholamines include dopamine, epinephrine, and norepinephrine and are synthesized from dietary tyrosine by the action of enzymes [42]. The biosynthesis and degradation of catecholamines occur in a wide range of cells, including the neurons of sympathetic nerves and the brain, adrenomedullary cells, endothelial cells, neutrophils, and mononuclear cells [43,44,45]. In human skin, catecholamine synthesis occurs in keratinocytes. Conversely, melanocytes also express mRNA and enzymes for the autocrine synthesis of norepinephrine but not epinephrine [7,42]. In human melanocytes, α-1-AR may be important in the reaction to norepinephrine, but melanin synthesis is also influenced by functional β_2_-AR signaling [7,8]. Interestingly, patients with vitiligo have increased β_2_-AR density in keratinocytes [46]. Increased norepinephrine levels are found in the urine and plasma of patients with non-segmental vitiligo, implying that catecholamine metabolism may be associated with the development and progression of vitiligo [47]. Moreover, in addition to classical stress neurotransmitters, melanocytes produce neuropeptides and hormones, such as corticotropin-releasing factor and proopiomelanocortin. This production is stimulated by UVR and other agents that act within the skin neuroendocrine system [48]. Therefore, the action of catecholamines and melanocytic function are closely related in a variety of complex pathways. Furthermore, carvedilol could interfere with the chemical reactions in the melanogenic pathway and this possibility should be further studied. Carvedilol may have advantages as it has simultaneous anti-inflammatory effects, as most hyperpigmentary disorders are PIH clinically or sub-clinically in dark-skinned patients. Since the permeation of carvedilol through the skin has been studied both in vitro and ex vivo, carvedilol can be developed as a topical whitening agent in the future [49,50,51].

Systemic carvedilol administration can cause bradycardia, dizziness, hypotension, headaches, and light-headedness. The topical application of carvedilol does not usually cause systemic symptoms; however, we should pay careful attention to these symptoms when applying it to patients with defective skin barriers, such as those with atopic dermatitis. Moreover, eczema, pruritus, and lichenoid eruption are reported in rare cases when taking carvedilol. These dermatological side effects as well as contact dermatitis should also be taken into account when applying carvedilol as a topical whitening agent.

In conclusion, we showed that carvedilol effectively reduced melanogenesis in human melanocytes and ex vivo human skin by inhibiting the cAMP/CREB/MITF pathway, which suggests its potential use as an effective whitening agent. Further investigation of the functional involvement of the adrenergic receptor by carvedilol in human melanocytes should follow.

## 4. Materials and Methods 

### 4.1. Materials

Carvedilol, 3,4-dihydroxy-L-phenylalanine (L-DOPA), cholera toxin (CT), and 12-O-tetradecanoylphorbol-13-acetate (TPA) were purchased from Sigma-Aldrich Co. (St. Louis, MO, USA). Dulbecco’s Modified Eagle’s Medium (DMEM) and Dulbecco’s phosphate-buffered saline were purchased from WelGENE (Daegu, Korea). Fetal bovine serum (FBS), antibiotic-antimycotic, and trypsin-EDTA were purchased from Gibco (Grand Island, NY, USA). Medium 254 (Cascade Biologics, Portland, OR, USA) and FSK ([3R-(3α,4aβ,5β,6β,6aα,10α,10aβ,10bα)]-5-(Acetyloxy)-3-ethenyldodecahydro-6,10,10b-trihydroxy-3,4a,7,7,10a-pentamethyl-1H-naphtho[2,1-b]pyran-1-one) were purchased from Tocris Bioscience (Bristol, UK).

### 4.2. Cell Lines and Cell Culture

Primary NHMs obtained from Invitrogen (Carlsbad, CA, USA) were maintained in Medium 254 (Thermo Fisher, Waltham, MA, USA) supplemented with Human Melanocyte Growth Supplement (Thermo Fisher). Mel-ab cells, a mouse-derived spontaneously immortalized melanocyte cell line, were obtained from the Korean Cell Line Bank (KCLB, Seoul, Korea) and maintained in DMEM supplemented with 10% FBS, penicillin-streptomycin, 100 nM TPA, and 1 nM CT. All cells were routinely maintained at 37 °C in a humidified environment of 5% CO_2_.

### 4.3. Antibodies and Western Blots

The cells were washed once with cold PBS and lysed in protein lysis buffer (1% SDS in 10 mM Tris and 5 mM EDTA, pH 7.4), followed by incubation at 98 °C for 10 min. The protein samples were separated by 8% SDS-polyacrylamide gel electrophoresis, blotted onto nitrocellulose membranes (GE Healthcare Life Sciences, Chicago, IL, USA), and then blocked with Tris-buffered saline containing 0.5% Tween 20 and 5% BSA, and subjected to immunoblotting. Tyrosinase and TRP-1 antibodies were purchased from Santa Cruz Biotechnology (Dallas, TX, USA), and MITF was purchased from Abcam (Cambridge, UK). α-tubulin (Gentex, Holland, MI, USA) was used as an internal loading control.

### 4.4. Melanin Content

The cytotoxic effect of carvedilol was evaluated using the Ez-Cytox Cell Viability Assay Kit (Dogen-Bio Co., Ltd., Seoul, Korea) in accordance with the manufacturer’s instructions.

Mel-Ab cells and NHMs were seeded in six-well plates at a density of 6 × 10^5^ and 3 × 10^5^ cells/well, respectively. Cells were treated with carvedilol, as shown in the figures, for 3 or 5 days (d). Before measuring the melanin content, the cells were observed under a phase contrast microscope and photographed (Olympus, Tokyo, Japan). Cells were dissolved in 550 µL of 1 N NaOH at 100 °C for 30 min and centrifuged at 13,000 rpm for 5 min. The absorbance of the supernatants was measured at 405 nm by a microplate reader. The intracellular melanin content was presented as a percentage relative to the cells’ untreated control. Arbutin (100 mg/mL) was used as a positive control.

### 4.5. Cellular Tyrosinase Activity

The tyrosinase activity was evaluated by measuring the rate of dopachrome formation of L-DOPA. After incubation with carvedilol, the cells were washed in ice-cold PBS and lysed in tyrosinase lysis buffer (phosphate buffer, pH 6.8, containing 1% Triton X-100) with repeated freeze/thaw cycles. The lysates were clarified by centrifugation at 15,000 rpm at 4 °C for 10 min. After quantifying the protein levels of the lysate and adjusting the protein concentrations with lysis buffer, 90 µL of supernatant mixed with 10 µL of 10 mM L-DOPA in tyrosinase lysis buffer was incubated at 37 °C. Cellular tyrosinase activity was measured by reading the absorbance at 475 nm using a microplate reader every 10 min for at least 1 h. Arbutin (100 mg/mL) was used as a positive control agent.

### 4.6. Immunohistochemical Analysis

Paraffin-embedded human skin tissues were cut into 6-μm-thick sections and stained with Melan-A (Novocastra, Newcastle, UK), Fontana-Masson kits (ID labs, London, ON, Canada), and HMB45 (Santa Clara, CA, USA), according to the manufacturers’ instructions. The melanin index was determined by measuring the percentage of stained area to the total tissue area using ImageJ 1.52a software (National Institutes of Health, Bethesda, MD, USA).

### 4.7. Statistical Analysis

The data are presented as the means ± standard error of the mean (S.E.M.), and statistical significance was determined by an unpaired Student’s *t*-test using GraphPad Prism5 software (San Diego, CA, USA). In this study, *p* < 0.05, *p* < 0.01, and *p* < 0.001 were considered statistically significant and are represented by *, **, and ***, respectively.
